# Identification of Redox and Glucose-Dependent Txnip Protein Interactions

**DOI:** 10.1155/2016/5829063

**Published:** 2016-06-29

**Authors:** Benjamin J. Forred, Skyla Neuharth, Dae In Kim, Michael W. Amolins, Khatereh Motamedchaboki, Kyle J. Roux, Peter F. Vitiello

**Affiliations:** ^1^Children's Health Research Center, Sanford Research, Sioux Falls, SD 57104, USA; ^2^Proteomics Facility, Sanford Burnham Prebys Medical Discovery Institute, La Jolla, CA 92037, USA; ^3^Department of Pediatrics, Sanford School of Medicine, The University of South Dakota, Sioux Falls, SD 57105, USA

## Abstract

Thioredoxin-interacting protein (Txnip) acts as a negative regulator of thioredoxin function and is a critical modulator of several diseases including, but not limited to, diabetes, ischemia-reperfusion cardiac injury, and carcinogenesis. Therefore, Txnip has become an attractive therapeutic target to alleviate disease pathologies. Although Txnip has been implicated with numerous cellular processes such as proliferation, fatty acid and glucose metabolism, inflammation, and apoptosis, the molecular mechanisms underlying these processes are largely unknown. The objective of these studies was to identify Txnip interacting proteins using the proximity-based labeling method, BioID, to understand differential regulation of pleiotropic Txnip cellular functions. The BioID transgene fused to Txnip expressed in HEK293 identified 31 interacting proteins. Many protein interactions were redox-dependent and were disrupted through mutation of a previously described reactive cysteine (C247S). Furthermore, we demonstrate that this model can be used to identify dynamic Txnip interactions due to known physiological regulators such as hyperglycemia. These data identify novel Txnip protein interactions and demonstrate dynamic interactions dependent on redox and glucose perturbations, providing clarification to the pleiotropic cellular functions of Txnip.

## 1. Introduction

Thioredoxin-interacting protein (Txnip/VDUP1/TBP-2) was originally discovered as a vitamin D3-inducible gene [1] but has gained recent interest for being involved in diabetes, hyperlipidemia, carcinogenesis, cardiac function, angiogenesis, and inflammation [2]. Structurally designated as part of the *α*-arrestin family, Txnip contains two aminoterminal SH3-binding domains while the carboxyl-terminus contains two PPxY motifs and three SH3 domains [3]. Txnip is involved in several prominent biological processes including proliferation, fatty acid and glucose metabolism, inflammation, and apoptosis [2]. A nonsense mutation in* Txnip* results in lipid and cholesterol accumulation due to reduced TCA cycle activity in the HcB-19 mouse strain [4]. Txnip also plays a major role in glucose homeostasis. The Txnip promoter contains several carbohydrate response elements (ChoRE) and Txnip is one of the most highly upregulated genes in pancreatic *β*-cells in response to hyperglycemia [5, 6]. As part of a negative-feedback loop, Txnip inhibits glucose uptake but also promotes caspase-3 cleavage, contributing to glucose-dependent *β*-cell death [7]. In addition, Txnip also regulates proinflammatory gene expression by inflammasome activation via NLRP3 binding [8]. Although we do not understand precise mechanisms governing differential Txnip signaling, it is clear that several of these pathways are linked by alterations in redox homeostasis.

Txnip is a unique target for redox perturbations since it is the only *α*-arrestin with a thioredoxin-binding domain [9]. Thioredoxins have vicinal thiols in their catalytic sites (CXXC) to facilitate redox signaling by regulating reversible cysteine oxidations of protein substrates [10]. In fact, genetic deletion of either thioredoxin-1 (Trx1, predominantly cytosolic) or thioredoxin-2 (Trx2, mitochondrial) results in embryonic lethality [11, 12]. Txnip is the only known endogenous inhibitor of both Trx1 and Trx2 activity [7, 13, 14]. Txnip forms an intermolecular disulfide via C247 to sequester and inhibit thioredoxins. Although cellular redox status is an important mediator of Txnip signaling, there are also redox-independent mechanisms to consider. Txnip also participates in a negative-feedback loop inhibiting glucose uptake; however, this inhibitory activity is retained by a cysteine to serine mutation incapable of binding thioredoxins. In this case, Txnip^C247S^ retains glucose uptake inhibitory activity in adipocytes and fibroblasts [15].

Because of its diverse array of functions, Txnip has been considered a novel candidate drug target for diabetes and cardiac ischemia-reperfusion injury. In fact, oral administration of verapamil, a calcium channel blocker, reduced murine Txnip expression and glucose-mediated apoptosis in *β*-cells [16]. Because of antiproliferative functions, Txnip silencing detected in numerous cancers is thought to be an important tumor-initiating event [17]. There is very little known about how impeding or reactivating Txnip expression influences downstream signaling pathways. To understand myocardial Txnip signaling during cardiac ischemia-reperfusion injury, Yoshioka et al. performed multiplex polony analysis of gene expression and proteomic profiling of* Txnip*-deficient mouse hearts to identify disrupted expression of genes functioning in mitochondrial metabolism [18]. However, since none of the reported mitochondrial enzymes were known to directly interact with Txnip, the molecular mechanisms underlying these cellular physiologies are not known.

To understand the pleiotropic cellular functions of Txnip, we used a proteomic approach to identify dynamic Txnip protein interactions in response to redox and glucose perturbations. Txnip fused to a promiscuous biotin ligase (BioID-Txnip) was expressed in HEK293 cells for biotinylation and affinity purification of interacting proteins [19, 20]. Subsequent proteomic analysis identified 31 Txnip protein interactions, only one of which was experimentally validated in a prior study. Many of the interactions were redox-dependent since protein binding was disrupted by mutating a critical cysteine (C247S) in Txnip known to facilitate binding to Trx1 [21]. Furthermore, many interactors were glucose-dependent and induced by hyperglycemic culture conditions. These data identify a large number of novel Txnip protein interactions which have the capacity to mediate differential signaling in response to changes in cellular redox and glucose concentration.

## 2. Materials and Methods

### 2.1. Cell Culture and Treatment

HEK293 cells (ATCC) were cultured in 5% CO_2_ at 37°C in high glucose (25 mM) DMEM with 10% fetal bovine serum, 50 U/mL penicillin, 50 *μ*g/mL streptomycin, and 20 *μ*g/mL gentamycin. For glucose sensitivity experiments, cells were acclimated in DMEM containing low glucose (5.5 mM) for a minimum of three passages and were treated with media supplemented with 5.5, 10, 15, 20, or 25 mM glucose for 24 hours before processing.

### 2.2. Generation of Stable BioID-Txnip Cell Lines

Myc-BirA^*∗*^-Txnip was generated by first generating Myc-BirA^*∗*^ by PCR from the pcDNA3.1 mycBioID plasmid [20]. PCR products were digested and ligated into pIRES2-EGFP (Clontech). Next, pCR4-TOPO-Txnip (Thermo Fisher Scientific) was used as template to amplify Txnip which was ligated in frame with Myc-BirA^*∗*^ in pIRES2-EGFP. Subsequently, Myc-BirA^*∗*^-Txnip^C247S^ was generated by site-directed mutagenesis (Agilent Technologies). Plasmids were linearized and transfected into HEK293 cells using Lipofectamine 2000 (Thermo Fisher Scientific). Cells were selected in 200 *μ*g/mL hygromycin and stable clones were selected based on EGFP fluorescence visualized with an Olympus IX71 inverted epifluorescent microscope.

### 2.3. SDS-PAGE and Immunoblot

As previously described [22], cell lysates were diluted in Laemmli buffer, separated by polyacrylamide gel electrophoresis (SDS-PAGE), and transferred to PVDF membranes. Membranes were blocked in 5% nonfat dry milk before incubating overnight at 4°C in rabbit anti-Txnip (1 : 1,000, Thermo Fisher Scientific), rabbit anti-myc (1 : 10,000, Abcam), or rabbit anti-*β*-actin (1 : 1,000, Sigma Aldrich). Blots were incubated with HRP-conjugated anti-rabbit secondary antibodies (1 : 5,000, Southern Biotech) or HRP-conjugated streptavidin (1 : 40,000, Invitrogen) for 1 hr at 25°C. Immune complexes were detected by chemiluminescence and images were captured and analyzed using a UVP bioimaging system.

### 2.4. Immunocytochemistry

Cells were cultured on 15 mm coverslips coated with poly-D-lysine in 12-well plates. Cells were washed with 1x PBS and fixed for 20 min with 3% paraformaldehyde (0.2 M phosphate buffer pH 7.3, 11% sucrose, and 0.1% Triton X-100). Cells were incubated for 30 min in blocking buffer (5% goat serum, 15 *μ*M BSA, 0.5% Triton X-100, and 0.05% sodium azide in 1x PBS) before overnight incubation with primary antibodies (1 : 1,000) at 4°C. Alexa Fluor-conjugated anti-secondary antibodies or streptavidin was incubated (1 : 2,000, Thermo Fisher Scientific) for 1 hr at 25°C and nuclei were counterstained with 0.5 *μ*g/mL DAPI in 1x PBS. Coverslips were mounted on slides and cells were visualized using a Nikon Eclipse 90i fluorescent microscope.

### 2.5. Affinity Capture of Biotinylated Proteins

Cells were cultured for 24 hrs in DMEM containing either 5.5 mM or 25 mM glucose supplemented with 50 *μ*M biotin. After washing three times with 1x PBS, cells were lysed at room temperature in 1 mL lysis buffer (50 mM Tris, pH 7.4, 500 mM NaCl, 0.4% SDS, 1 mM DTT, and 1x complete protease inhibitor [Roche]). Triton X-100 was supplemented to a 2% final concentration and sonicated two times using the Branson Sonifier 250 at 30% duty cycle and an output level of 3 for 1 min. An equal volume of 4°C 50 mM Tris (pH 7.4) was added before additional sonication and centrifugation at 16,000 rpm at 4°C. Supernatants were incubated with 600 *μ*L Dynabeads (MyOne Streptavidin C1, Invitrogen) overnight at 4°C with an end-over-end rotator. Beads were collected and washed two times for 8 min at 25°C in 1 mL wash buffer 1 (2% SDS in ddH_2_O). This was repeated once with wash buffer 2 (0.1% deoxycholate, 1% Triton X-100, 500 mM NaCl, 1 mM EDTA, and 50 mM Hepes, pH 7.5), once with wash buffer 3 (250 mM LiCl, 0.5% NP-40, 0.5% deoxycholate, 1 mM EDTA, and 10 mM Tris, pH 8.1), and two times with wash buffer 4 (50 mM Tris, pH 7.4, and 50 mM NaCl). After the final wash, 10% of the sample was reserved for immunoblot analysis. To this end, 50 *μ*L of Laemmli SDS-sample buffer saturated with biotin was added to the 10% saved sample and heated at 98°C. For the larger scale mass-spectrometry analysis, 90% of the sample was reserved in in 50 mM NH_4_HCO_3_.

### 2.6. BioID, On-Bead Protein Digestion, and Identification by 1D LC-MS/MS

Large-scale BioID pull-downs for MS analysis were performed using the 90% of sample resulting from affinity capture with streptavidin-conjugated magnetic beads. Sample volume was adjusted to 200 *μ*L with 50 mM ammonium bicarbonate. 4 *μ*L of 0.5 M tris(2-carboxyethyl)phosphine was added to 200 *μ*L of the beads-proteins suspension mix, and proteins were reduced at 40°C for 30 min. Next, 8 *μ*L of 0.5 M iodoacetamide was added, and proteins were alkylated at room temperature for 30 min, in the dark. MS-grade trypsin (Promega) was added (1 : 20 ratio) for overnight digestion at 37°C using an Eppendorf Thermomixer at 700 rpm. Peptides were separated from magnetic beads by centrifugation and a GE Healthcare MagRack and were transferred to a new tube. Formic acid was added to the peptide solution to 2% final concentration, followed by desalting by Microtrap (Thermo Fisher Scientific) and then online analysis of peptides by high-resolution, high-mass accuracy liquid chromatography tandem MS (LC-MS/MS) consisting of a Michrom HPLC, a 15 cm Michrom Magic C18 column, a low-flow ADVANCED Michrom MS source, and a LTQ-Orbitrap XL (Thermo Fisher Scientific). A 120 min gradient of 10–30% B (0.1% formic acid, 100% acetonitrile) was used to separate the peptides. The total LC time was 140 min. The LTQ-Orbitrap XL was set to scan precursors in the Orbitrap followed by data-dependent MS/MS of the top 10 precursors. Raw LC-MS/MS data were submitted to Sorcerer Enterprise (Sage-N Research Inc.) for protein identification against the ipi.HUMAN.vs.3.73 protein database. Differential search included 16 Da for methionine oxidation, 57 Da for cysteines to account for carboxyamidomethylation, and 226 Da for biotinylation of lysine. Search results were sorted, filtered, statically analyzed, and displayed using PeptideProphet and ProteinProphet (Institute for Systems Biology). The minimum Trans-Proteomic Pipeline (TPP) probability score for proteins was set to 0.95 to ensure a TPP error rate lower than 0.01. The relative abundance of each of the identified proteins in different samples was analyzed by QTools, an open-source tool developed in-house for automated differential peptide/protein spectral count analysis. Proteins from the HEK293 control sample and common BioID background proteins were eliminated from the results to minimize noise.

### 2.7. Bioinformatics Analysis

Proteins with less than three spectral counts or common mass-spec background proteins including keratins, histones, and ribosomal proteins were removed due to the lack of confidence. Proteins identified from parental HEK293 cells were used to remove false positive candidates from BioID-Txnip samples. However, proteins whose relative percentage of total spectral counts was threefold more than in the HEK293 parental controls were also considered candidates. Primary subcellular localization of proteins was determined based on information from UniProt and the Human Protein Atlas. Functional categorization was based on information from UniProt and NCBI:Gene reports. Proteomic data was analyzed for predicted molecular and cellular functions and referenced against experimentally observed protein-protein networks by Ingenuity Pathway Analysis (IPA) software.

## 3. Results

We utilized the novel BioID system as an unbiased proteomic approach to identify Txnip protein interactions [20]. A promiscuous biotin ligase from* E. coli* (BirA^*∗*^) with an aminoterminal myc epitope was fused to human Txnip (henceforth called BirA^*∗*^-Txnip). We chose to generate an aminoterminal fusion protein because BirA^*∗*^ is similarly sized to green fluorescence protein (GFP, ~35 and 27 kDa, resp.) and ectopic expression of GFP-Txnip retained proper localization [23] as well as apoptotic function [24]. By mapping biotinylation of known nucleoporin complexes, it is suggested that BirA^*∗*^ has a labeling radius ~10 nm [19]. As such, this system was used to biotinylate only those proteins within close enough physical proximity highly likely to facilitate protein-protein interactions.

**Figure 1:**
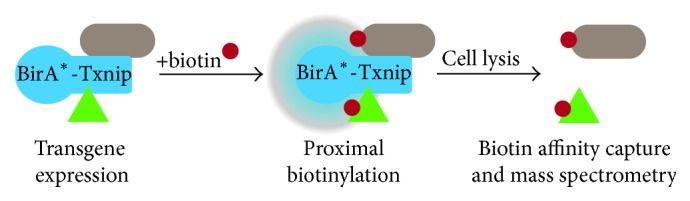
BioID system for identification of Txnip interacting proteins. Myc-BirA^*∗*^ was fused to the aminoterminus of Txnip (BirA^*∗*^-Txnip) to screen for protein interactions. BirA^*∗*^ catalyzes a two-step reaction: first, generation of reactive biotinyl-AMP from biotin and ATP, and second, the attachment of that biotinyl-AMP to a specific lysine on an interacting/proximal protein. Streptavidin beads are used to affinity-purify biotinylated proteins, which then are analyzed by mass spectrometry.

**Figure 2:**
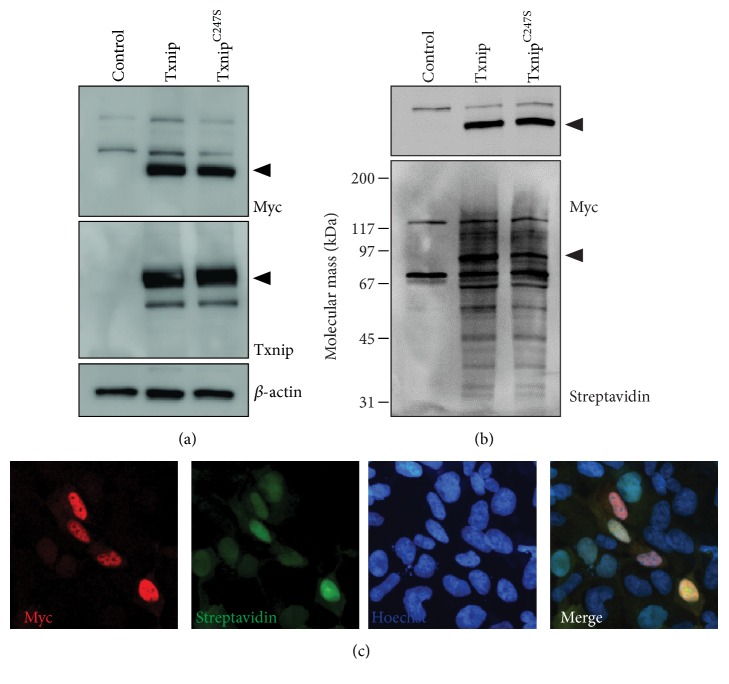
BirA^*∗*^-Txnip expression, activity, and localization. (a) Protein lysates from HEK293 parental (control) or cells stably expressing either BirA^*∗*^-Txnip or BirA^*∗*^-Txnip^C247S^ were separated by SDS-PAGE and analyzed via immunoblot for transgene expression using myc and Txnip with *β*-actin as a loading control. Arrowheads indicate the expected size of the BioID transgenes. Cells were pulsed with biotin for 24 hrs prior to lysis and biotinylated proteins were detected by (b) affinity purification and SDS-PAGE/immunoblot and (c) immunocytochemistry. The BioID transgene was detected with myc and biotinylated proteins were detected with streptavidin. Images are representative of 3 biological replicates.

HEK293 cells stably expressing BirA^*∗*^-Txnip or a cysteine mutant, BirA^*∗*^-Txnip^C247S^, which is incapable of binding thioredoxins [21], were cultured in excess biotin to label endogenous proximal proteins (Figure 1). Cells had very similar expression levels and biotinylation signals with either BirA^*∗*^-Txnip or BirA^*∗*^-Txnip^C247S^ (Figures 2(a) and 2(b)). Consistent with prior reports for endogenous Txnip, the BirA^*∗*^-Txnip transgene localized primarily to the nucleus with faint cytosolic staining (Figure 2(c)) [23, 25]. BirA^*∗*^-Txnip activity is noted due to colocalization of the transgene (anti-myc) with biotinylated proteins (streptavidin). A similar localization pattern was detected with BirA^*∗*^-Txnip^C247S^ (data not shown).

To identify Txnip interacting proteins, cells were cultured in media supplemented with 50 *μ*M biotin and subsequently lysed under stringent conditions to solubilize proteins and disrupt protein-protein interactions. Biotinylated proteins were affinity-purified with streptavidin-conjugated paramagnetic beads from lysates of HEK293 parental (control), BirA^*∗*^-Txnip, and BirA^*∗*^-Txnip^C247S^ cells (Figure 2(b)). After extensive washing, biotinylated proteins that bound the streptavidin beads were digested and analyzed by 1D LC-MS/MS. In total, we detected 31 proteins unique to the BirA^*∗*^-Txnip pull-down that were identified in at least 2 of 3 independent BioID trials (Table 1). From this list, 30 of the proteins were novel Txnip interactions not previously identified through experimental validation. ITCH (E3 ubiquitin-protein ligase Itchy homolog), a known Txnip interactor, was identified as an E3 ubiquitin ligase for Txnip. This interaction was previously shown to be governed by the PPxY motifs of Txnip with WW domains of ITCH [3]. Consistent with BirA^*∗*^-Txnip localization, the majority of proteins identified (19 of 31) primarily localized to the nucleus. However, proteins known to localize to the cytoplasm, plasma membrane, ER, and vesicles were also detected. Txnip protein interactors had a diverse array of molecular functions, with a majority serving as chaperones or in the regulation of chromatin structure and gene expression (Table 1).

**Figure 3:**
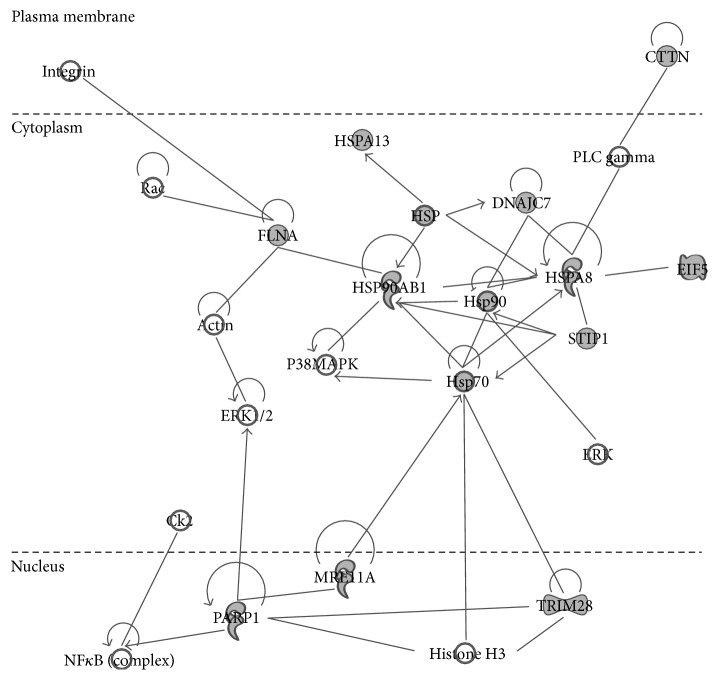
IPA network analysis of Txnip proteins identified by BirA^*∗*^-Txnip. BirA^*∗*^-Txnip interactions analyzed by IPA identifying 14 proteins (shaded in gray) included the drug metabolism, endocrine system development and function, and lipid metabolism network. IPA analysis was restricted to only include experimentally observed protein interactions.

Proteins identified by BirA^*∗*^-Txnip were analyzed via pathway analysis using IPA software. Txnip function is already associated with alterations in cell proliferation and lipid metabolism, two of the IPA predicted molecular and cellular functional categories (Table 2). After filtering out nonexperimentally validated interactions, two protein networks aligned with our proteomic data set: (i) drug metabolism, endocrine system development and function, and lipid metabolism (Figure 3) and (ii) cell death and survival, cellular development, and embryonic development.

Since Txnip^C247^ was previously shown to be critical for thioredoxin binding, we performed a comparative proteomic analysis between cells expressing BirA^*∗*^-Txnip and BirA^*∗*^-Txnip^C247S^ to identify redox-sensitive protein interactions. 17 of the 31 Txnip protein interactions were lost following the cysteine to serine mutation (Table 1). Interestingly, interactions with all 6 chaperones (HSP90AB1, HSP1A/1B, HSPA13, DNAJC7, HSPA8, and STIP1) were lost in BirA^*∗*^-Txnip^C247S^. However, this is not surprising as heat shock proteins are known to harbor highly reactive cysteines [26, 27]. It is also not surprising that many of the protein interactions were sustained and are likely to occur through the multiple PPxY and/or SH3 domains in Txnip. For example, ITCH interacts through a PPxY domain near the Txnip carboxyterminus and was detected in BirA^*∗*^-Txnip^C247S^ [3]. Together, these data demonstrate the utility of BioID to identify novel Txnip protein interactions, many of which are dependent on C247 thiol reactivity.

**Figure 4:**
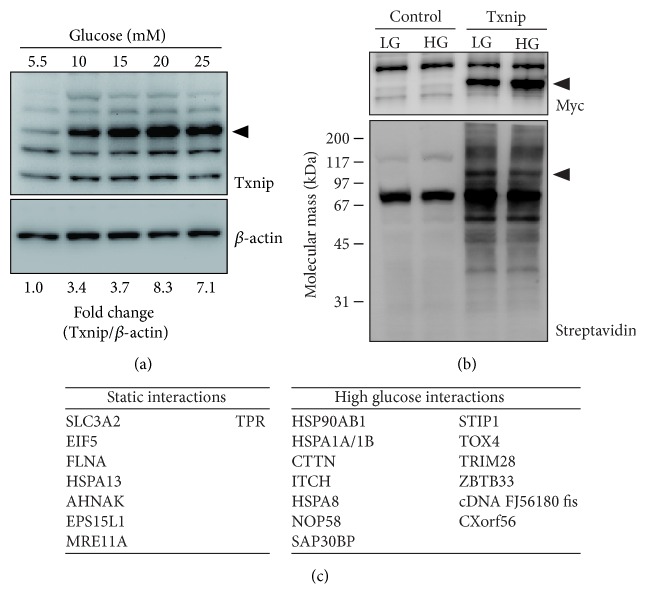
Glucose-dependent Txnip expression and protein interactions. HEK293 parental cells acclimated to culture in low glucose media (5 mM) were treated for 24 hrs with increasing glucose concentrations (5, 10, 15, 20, and 25 mM) and protein lysates were analyzed by SDS-PAGE/immunoblot for Txnip with *β*-actin as a loading control. The arrowhead indicates the expected size of Txnip and densitometry values were determined by the pixel density ratio for Txnip/*β*-actin. (b) HEK293 parental (control) or cells stably expressing BirA^*∗*^-Txnip were cultured in low glucose (5.5 mM, LG) or high glucose (25 mM, HG) for 24 hrs and then pulsed with biotin. Biotinylated proteins were detected by SDS-PAGE/immunoblot and affinity purification. (c) Affinity purified biotinylated proteins were digested and analyzed by mass spectrometry. BirA^*∗*^-Txnip interactors were classified as static (identified in both LG and HG) or high glucose (only identified in HG). Images are representative of 3 biological replicates.

Txnip gene expression is upregulated in response to glucose [5]; therefore, we hypothesized that Txnip protein interactions may also be glucose-dependent. All prior experiments were performed using media supplemented with 25 mM glucose. To investigate Txnip glucose-inducibility, HEK293 parental cells were acclimated to low glucose media (5.5 mM) for at least three passages. Endogenous Txnip expression was increased in cells cultured in hyperglycemic media (Figure 4(a)). Augmented Txnip protein expression in HEK293 cells was robust with a 7.1-fold increase following culture in media supplemented to 25 mM glucose. Maximal Txnip expression was detected after culturing cells for 24 hrs in 25 mM glucose (data not shown). Cells expressing BirA^*∗*^-Txnip were similarly acclimated to low glucose conditions and were pulsed with biotin following a switch to media supplemented with high glucose (25 mM). There were no gross changes in the biotinylation signal due to glucose culture conditions via antistreptavidin immunoblot (Figure 4(b)). However, mass spectrometry of affinity purified lysates identified Txnip interactions that were independent of glucose concentration (identified in both conditions) or dependent on increased glucose in the media (only identified in 25 mM glucose culture) (Figure 4(c)). These data suggest that the list of Txnip interactions is dynamic and depends upon the glucose concentration in the culture media.

## 4. Discussion

While there is much interest in the physiological relevance of Txnip, there is limited knowledge of the underlying molecular interactions supporting Txnip-dependent changes in cellular function. We sought to identify novel Txnip protein interactions which may govern differential signaling pathways responsible for pleiotropic cellular functions. Using BioID as an approach to identify protein interactions is advantageous over common methods such as a yeast-2-hybrid screen (not typically performed in human cells) and endogenous immunoprecipitation (only detecting static interactions). An alternative proximity-based labeling system, APEX or APEX2, is not amenable for identification of redox-dependent pathways since it requires cell treatment with hydrogen peroxide to generate biotin-phenoxyl radicals and appears limited to compartmental proteomics [28, 29]. There are several considerations using HEK293 cells for proteomic analysis. Use of an immortalized tumor cell line may not mirror physiological conditions due to imbalances in redox homeostasis [30] and altered metabolic preferences [31]. This is more critical given known roles of Txnip in response to redox perturbations and glucose and lipid metabolism. Txnip protein interactions may be cell-specific based on unique expression or regulatory mechanisms in specialized cells such as pancreatic *β*-cells [5] or cardiomyocytes [18]. However, Txnip expression is relatively ubiquitous with the exception of the central nervous system; therefore, use of HEK293 cells as a prototypical cell conduit for these untargeted proteomic studies was justified.

Our data support that dynamic protein interactions may facilitate unique signaling pathways, accounting for the pleiotropic cellular functions of Txnip. We propose two likely mechanisms that influence protein binding partners: (i) subcellular trafficking and localization and (ii) differential posttranslational modifications. As expected, Txnip-BirA^*∗*^ was predominantly localized in the nucleus with faint detection of cytosolic staining (Figure 2(c)). Depending on the oxidative stimulus, Txnip has been demonstrated to traffic from the nucleus. For example, Txnip accumulates in the cytosol and complexes with the NLRP3 inflammasome following hydrogen peroxide treatment [8]. Upon glucose stimulation, Txnip shuttles to the mitochondria and relieves Trx2 inhibitory binding to apoptosis signal-regulating kinase (ASK1), promoting glucose-dependent *β*-cell apoptosis [7]. Although hyperglycemic treatment increased Txnip expression (Figure 4(a)), neither endogenous Txnip nor the BioID transgene (or any biotinylated proteins) was detected in mitochondria (data not shown). While it is already known that thiol oxidation of C247 facilitates Trx1 and Trx2 binding, Liu et al. recently described a novel tyrosine phosphorylation of the PPxY motifs that influences ITCH binding [32]. Txnip PPxY phosphorylation decreased ITCH binding and promoted PTPN11 (tyrosine-protein phosphatase nonreceptor type 11) interactions, resulting in c-Src tyrosine-protein kinase activation. Further understanding of the relationship between Txnip localization, posttranslational modifications, differential protein interactions, signaling pathways, and functional consequences is necessary to refine therapeutic strategies targeting Txnip [16].

Currently, 15 proteins have been validated to directly interact with Txnip through experimental means. This includes Trx1 [13], NLRP3 [8], HDAC1 [33], and p53 [34]. We identified 31 proteins biotinylated by BirA^*∗*^-Txnip but only one, ITCH, was previously described as a Txnip binding partner [3]. While it is possible that many of these interactions exhibit cell specificity, there are technical and functional differences likely accounting for dissimilarities. Since BirA^*∗*^ has a labeling radius of ~10 nm [19], it is important to consider that spatial constrictions may exclude protein biotinylation in addition to physical interactions restricted by BirA^*∗*^ fusion. For example, Txnip is mainly comprised of *β*-strands that form an elongated S-shaped domain divided into amino- and carboxyterminal domains [9]. Trx1 C35 interacts exclusively with Txnip C247 of the carboxyterminal domain, which may be spatially restricted to aminoterminal BirA^*∗*^. However, 15 of 31 Txnip interactions were not detected in BirA^*∗*^-Txnip^C247S^. It is difficult to speculate if these dynamic interactions were a direct consequence of loss of binding at C247 or if this was a result of Txnip structural alterations in disulfide bond switching [9]. A crystal structure including the C-terminal PPxY motifs (amino acid positions 331 and 375) has not been determined although our proteomic data identifying ITCH suggest that the Txnip carboxyterminal tail is flexible and accessible to aminoterminal BirA^*∗*^.

It was not surprising that IPA analysis of Txnip protein interactions identified cellular growth and proliferation as a major predicted functional pathway based on known interactions (Table 2).* Txnip*-deficient fibroblasts proliferate more rapidly than wild-type counterparts [35] and the HcB-19 strain (spontaneous Txnip mutation) has increased incidence of hepatocellular carcinomas [36]. Txnip has been considered a tumor suppressor because of suppressed expression in a variety of cancers (reviewed by [17]). Therefore, there is high therapeutic interest in using small molecules to reactivate Txnip expression as an anticancer strategy [37]. One speculative growth pathway based on our proteomic data involves Poly ADP-ribose polymerase 1 (PARP1). Reduced expression of the cyclin-dependent kinase inhibitor, p27^Kip1^, correlated with loss of Txnip [35]. The Txnip:PARP1 axis (Table 1) may regulate p27^Kip1^ since PARP1 represses FOXO1-mediated p27^Kip1^ expression [38].

Another speculative pathway that may be linked to pleiotropic effects of Txnip involves heat shock proteins (HSP). HSP90 and HSPA1A/B (also known as HSP70) were exclusively detected during culture in high glucose (Figure 4(c)). This suggests that these chaperones play a signaling role during hyperglycemia and are not caused by supraphysiological exogenous gene expression. The HSP90:HSP70 chaperone machinery may regulate Txnip trafficking and/or turnover due to oxidative damage [39]. This hypothesis is supported by glucose-dependent interactions with the E3 ubiquitin ligase, ITCH, a likely negative-feedback response to promote Txnip proteasomal degradation following hyperglycemic gene induction. Conversely, Txnip interactions with HSP90:HSP70 may directly facilitate signaling pathways linked to pleiotropic cellular functions ascribed to Txnip. For example, HSP90 and HSP70 have cyclic function to regulate and enhance glucocorticoid receptor activation and function [40].

In conclusion, these studies identified novel Txnip protein interactions in response to redox and glucose perturbations which may have relevance for several cellular functions and pathologies. Since BioID relies on affinity purification of biotinylated targets, as opposed to organelle or bait purification, we demonstrate how this approach is advantageous for detection of dynamic, redox-dependent Txnip interactions occurring across different cellular compartments [41].

## Figures and Tables

**Table 1:** List of proteins identified by BirA^*∗*^-Txnip.

Protein name^1^	Gene ID	UniProt	Localization^2^	Molecular function^3^	C247S
Src substrate cortactin	CTTN	Q14247	Plasma membrane	Actin polymerization	Yes

Filamin-A, isoform 2	FLNA	Q60FE6	Cytoplasm	Actin polymerization	No

4F2 cell-surface antigen heavy chain, isoform 2	SLC3A2	P08195	Plasma membrane	Amino acid transport	Yes

Heat shock protein HSP 90-beta	HSP90AB1	P08238	Cytoplasm	Chaperone	No

Heat shock 70 kDa protein 1A/1B	HSPA1A/1B	P0DMV8	Cytoplasm	Chaperone	Yes

Heat shock 70 kDa protein 13	HSPA13	P48723	ER	Chaperone	Yes

DnaJ homolog subfamily C member 7	DNAJC7	Q99615	Cytoplasm	Chaperone	Yes

Heat shock cognate 71 kDa protein	HSPA8	P11142	Cytoplasm, nucleus	Chaperone	Yes

Stress-induced phosphoprotein 1	STIP1	P31948	Cytoplasm, nucleus	Chaperone	Yes

JmjC domain-containing histone demethylation protein 2C	JMJD1C	B7ZLC8	Nucleus	Chromatin structure	No

TOX high mobility group box family member 4	TOX4	O94842	Nucleus	Chromatin structure	Yes

YEATS domain-containing protein 2	YEATS2	Q9ULM3	Nucleus	Chromatin structure	Yes

Double-strand break repair protein MRE11A	MRE11A	B3KTC7	Nucleus	DNA repair	No

Poly ADP-ribose polymerase 1	PARP1	P09874	Nucleus	DNA repair	No

Epidermal growth factor receptor substrate 15-like 1	EPS15L1	A5PKY0	Vesicles	Metal binding	No

Nuclear mitotic apparatus protein 1	NUMA1	Q14980	Nucleus	Mitotic spindle formation	Yes

TPR nucleoprotein	TPR	P12270	Nucleus	Nuclear transport	No

E3 ubiquitin-protein ligase Itchy homolog	ITCH	Q96J02	Cytoplasm	Proteolysis	No

E2 ubiquitin-conjugating enzyme	UBE2O	Q9C0C9	Nucleus	Proteolysis	Yes

Thioredoxin-like protein 1	TXNL1	O43396	Cytoplasm	Redox	Yes

Neuroblast differentiation-associated protein AHNAK	AHNAK	Q09666	Nucleus	RNA binding	No

Nucleolar protein 58	NOP58	Q9Y2X3	Nucleus	RNA binding	No

U4/U6.U5 tri-snRNP-associated protein 1	SART1	O43290	Nucleus	RNA binding	Yes

Activity-dependent neuroprotector homeobox protein	ADNP	Q9H2P0	Nucleus	Transcription	Yes

SAP30-binding protein	SAP30BP	Q9UHR5	Nucleus	Transcription	No

Sex comb on midleg-like protein 2	SCML2	H0Y6S1	Nucleus	Transcription	No

Transcription intermediary factor 1-beta	TRIM28	Q13263	Nucleus	Transcription	Yes

Transcriptional regulator Kaiso	ZBTB33	Q86T24	Nucleus	Transcription	No

Eukaryotic translation initiation factor 5	EIF5	P55010	Cytoplasm	Translation	Yes

cDNA FLJ56180 fis	N/A	Q6ZNN8	Unknown	Unknown	No

UPF0428 protein CXorf56	CXorf56	Q9H5V9	Nucleus	Unknown	No

^1^Proteins were identified on at least 2 of 3 biological replicates.

^2^Primary localization based on information from UniProt and The Human Protein Atlas.

^3^Based on information from UniProt and NCBI:Gene reports.

**Table 2:** Predicted molecular and cellular functions of BirA^*∗*^-Txnip interacting proteins.

Molecular and cellular function	*p* value range	# molecules
Cellular growth and proliferation	3.12*e* ^−2^–1.70*e* ^−5^	19
Drug metabolism	8.12*e* ^−3^–6.36*e* ^−5^	5
Lipid metabolism	8.12*e* ^−3^–6.36*e* ^−5^	3
Small molecular biochemistry	3.60*e* ^−2^–6.36*e* ^−5^	5
Cellular assembly and organization	3.73*e* ^−2^–1.37*e* ^−4^	9
